# Prevalence and factors associated with lower limb amputation in individuals with type II diabetes mellitus in a referral hospital in Fortaleza, Ceará, Brazil: A hospital-based cross-sectional study

**DOI:** 10.1016/j.heliyon.2020.e04469

**Published:** 2020-07-18

**Authors:** Francisca Lesse Mary Teixeira Alves, Gabriel Zorello Laporta

**Affiliations:** a Setor de Pós-graduação, Pesquisa e Inovação, Centro Universitário Saúde ABC (FMABC), Fundação ABC, Santo André, São Paulo, Brazil; b Hospital Geral de Fortaleza (HGF), Secretaria de Estado da Saúde State, Fortaleza, Ceará, Brazil

**Keywords:** Diabetes, Insulin, Epidemiology, Public health, Quality of life, Disability, Endocrinology, Metabolism, Metabolic disorder

## Abstract

**Aims:**

To analyze the association between demographic, socioeconomic, clinical, epidemiological, and primary healthcare factors with the severity of lower limb amputations (LLAs) in individuals with type II diabetes mellitus (DM-II) at a reference hospital in Fortaleza, Ceará, in Northeast Brazil.

**Methods:**

A cross-sectional study was performed with a representative sample of individuals hospitalized with DM-II and the degree of LLA severity: (1) toes; (2) transmetatarsal or infrapatellar; (3) suprapatellar; (4) disarticulation or bilateral. Potentially associated factors with the outcome degree of amputation severity were identified in a semi-structured evaluation during hospitalization. The prevalence ratios of the degree of amputation severity as a function of associated factors were calculated with robust variance Poisson regression models.

**Results:**

The prevalence of high degree of severity in amputations (suprapatellar, with disarticulation or bilateral) was high in the total sample of 385 patients, revealing to be 49% (187/385). Prevalence ratios (PR) indicated a higher prevalence of DM-II amputation severity in patients who lacked of specific guidance on DM-II amputation in primary care (PR = 1.52, 95% CI: 1.05–2.21).

**Conclusions:**

LLAs in DM-II were associated with age above 67 years, male gender, cardiovascular disease, and low support for guidance at the primary healthcare level.

## 1 Introduction

Diabetes mellitus (DM) is one of the most prevalent chronic diseases worldwide, and an estimated 415 million people were diagnosed with DM in 2015 [1]. Increasing population aging associated with lifestyle changes contributes to the exponential increase in disease prevalence. Projections for 2030 and 2040 indicate increases of up to 50% in world prevalence with estimates of affected individuals between 439-642 million [2, 3, 4]. Brazil is in fourth position in the world in number of individuals with DM [5]. The Brazilian National Health Survey in 2013 showed a self-reported DM prevalence of 6% in the age group ≥18 years, and up to 20% in the 65 to 74 age group [6]. In the Northeast region of Brazil, Flor and Campos (2017) [7] identified a self-reported prevalence of 6% considering all age groups. DM accounted for 21% of all deaths reported in the Ceará State Epidemiological Bulletin of Non-Communicable Diseases (NCDs) in 2017 [8]. The mortality rate for DM recorded in *DATASUS* in 2017 in Ceará was 25 per 100,000 inhabitants [9].

Type 2 DM (DM-II) accounts for 90–95% of all DM cases and is a progressive metabolic disorder characterized by resistance to insulin effects and abnormal insulin production to maintain blood glucose levels [10, 11], presenting a complex and multifactorial etiology [12, 13, 14, 15]. The main chronic consequences of the disease are classified as: (1) macrovascular, especially coronary artery disease, cerebrovascular and peripheral vascular disease; (2) microvascular, characterized by vision damage (retinopathy), kidney disease (nephropathy) and neuronal injury (neuropathy) [11, 16, 17, 18, 19]. The latter represent the most common comorbidities: kidney disease, irreversible blindness and non-traumatic lower limb amputations.

Diabetic foot is a clinical disorder with sustained hyperglycemia-induced neuropathy and minor trauma (e.g., tight shoes, barefeet, acute foot injury, etc.). The result is ulceration in the foot region [20]. Diabetic foot syndrome involves pathological conditions, including: (1) neuropathy with loss of sensation; (2) peripheral arterial disease; (3) Charcot's neuroarthropathy; (4) reduced joint mobility; (5) foot ulceration and osteomyelitis resulting in abnormal biomechanical burden on the foot; (6) callus and ulcer formation. The combination and clinical worsening of these conditions can trigger lower limb amputations [14, 20]. These amputations are largely complex, disabling, costly and exacerbate healthcare costs [21, 22, 23, 24].

Prevention of about 90% of lower limb amputation (LLA) procedures could be ensured by screening through appropriate health surveys and health guidance [25, 26]. It is estimated that 30–50% of those who have had an amputation will require additional amputations within 1–3 years, and 74% will die within 5 years after the first amputation [21, 26, 27]. The repercussions of amputations are directly associated with the formation of new ulcers, loss of functional mobility, loss of autonomy, high rate of depression and decreased quality of life [13, 28].

The multifaceted complications of DM and its repercussions related to the extremity of the lower limb (such as diabetic foot and amputations) are increasing, with a wide impact on public health and need to be investigated so that strategies can be devised and indicators can be used to follow up these disorders, expanding the knowledge in clinical practice. In addition, a double odds ratio of hospital death was observed in a nationwide study on hospitalizations for DM for the inhabitants of the Northeast region [29]. A 4% (n = 67) proportion of individuals with diabetic foot was identified in an analysis of 1,631 patients treated at the emergency of a tertiary public hospital in Fortaleza/CE, of which 97% were treated with surgical procedure due to the severity degree of the injury [27].

The city of Fortaleza was chosen for this study because it contains a state-level hospital referral center with specialized care in medium and high complexities, responsible for clinical support to patients with DM. The overall objective of the study was to analyze the association between demographic, socioeconomic, clinical, epidemiological, and primary healthcare factors with the lower limb amputation severity in individuals with DM-II at a reference hospital in Fortaleza, Ceará, Northeast, Brazil. The specific objectives were: (1) to identify the prevalence of LLA severity degrees in DM-II; and (2) to analyze the prevalence ratios of LLA severity degrees in DM-II as a function of demographic, socioeconomic, clinical, epidemiological and primary healthcare.

## 2 Materials and methods

### 2.1 Study design

The study was conducted with a cross-sectional epidemiological design and a sampling plan defined by a reference hospital in the city of Fortaleza, Ceará, Northeast Brazil with a representative sample of individuals hospitalized with DM-II and lower limb amputation (LLA).

The study was approved by the Research Ethics Committee of the Fortaleza General Hospital (*HGF*) in Fortaleza, Ceará, Brazil, under Opinion No. 1,912,545, and CAE no. 64048317.8.0000.5040.

The study was conducted at Fortaleza General Hospital [30] (*HGF*; http://www.hgf.ce.gov.br; 3°44′21″ South 38°28′35″ West) [31]. This is the largest public hospital in the Unified Health System (*SUS*) network linked to the State Secretary of Health of Ceará. It is a reference in highly complex procedures with neurosurgery, transplantation, stroke care and other neurological, orthopedic, endocrine, high-risk obstetric, and specialized clinical treatments, among others. It is the main reference hospital for high complexity care in individuals with diabetes in the state of Ceará [30]. The *HGF* has 541 elective, emergency, and obstetric care beds, and adult and neonatal intensive care units. An average of 600 elective surgeries and 19,000 consultations are performed per month, and 210,000 laboratory tests are performed with more than 8,000 imaging exams [30].

### 2.2 Study size

The study population (sample) was selected based on: (1) the minimum sample size and (2) cumulative sampling.

The minimum sample size of 384 was estimated from the following sample calculation equation:

$$n=\;z_{\frac{\alpha }{2}}^{2}\frac{P\left(1-P\right)}{\varepsilon^{2}}=384$$

in which the following parameters were defined: (1) 95% confidence interval$\;z_{\frac{\alpha }{2}}^{2}=1.96^{2}$; (2) 50% prevalence of LLA severity by DM assumed *a priori* P=0.50; and (3) 5% tolerated error in estimating prevalence $\;\varepsilon^{2}=\;0.05^{2}$.

Sampling was cumulative, i.e. **all individuals who met the eligibility criteria** within the recruitment period (Feb. 2017–Apr. 2018) were selected until reaching the previously defined sample size (n = 384).

The sampling plan was probabilistic because all inhabitants of Fortaleza or neighboring municipalities (metropolitan region of Fortaleza) who met the eligibility criteria had the same *a priori* probability of being recruited for the study sample. This assumption is supported by the fact that the *HGF* public hospital is a reference in the region for the specialized and complex treatment of patients with DM and complications such as LLA. The accessible population of the study were *HGF* DM patients in the collection period from Feb. 2017–Apr. 2018.

### 2.3 Participants

Eligibility criteria were: (1) clinical diagnosis of type 2 DM (DM-II); and (2) lower limb amputation (LLA) surgical procedure.

All participants were admitted to the *HGF* during the study recruitment period in the emergency room, recovery room, and vascular ward. The selected individuals were invited to participate in the study after the amputation procedure, which consisted of an interview with evaluation and application of an evaluation instrument. All participants received information about the study objectives and procedures and signed the Informed Consent Form.

### 2.4 Variables

The exposure factor of the present study was DM (cause) and the LLA severity was the outcome (effect). The cause-and-effect measurement and associated factors were performed at a single time point. The performed analysis considered the outcome (amputation severity) as a function of factors associated with DM-II for estimates of prevalence ratios (i.e. measure of association). The outcome was represented by an ordinal qualitative variable corresponding to the degrees of LLA severity as complications of DM-II: outcome 0 - less severe (toe amputation); outcome 1 - severe (transmetatarsal and infrapatellar amputation); outcome 2 - very severe (suprapatellar amputation); and outcome 3 - extremely severe (disarticulation and bilateral amputation). Factors associated with DM-II and considered determinant for LLA are the independent or explanatory variables, as follows:

(1) Clinical (time since diagnosis, risk factors, medication use, associated diseases, complications and comorbidities);
(2) Sociodemographic characteristics (age, gender, education, city of residence);
(3) Information on self-care (glucometer use, daily glucose control, oral hypoglycemic use, insulin use); and
(4) Healthcare in the primary sector (prior knowledge of DM, frequency of primary care, guidance or specific information on amputation in DM in primary healthcare).

### 2.5 Data sources/measurement

Individuals who agreed to participate in the study underwent an evaluation during hospitalization and clinical follow-up. The order of the evaluations occurred according to the hospitalization period. The evaluation began after hemodynamic stabilization, with an average duration of 40 min and at intervals whenever the patient showed tiredness or indisposition. A structured interview was additionally performed or supplemented with the caregiver in charge when the patient was sedated, with endotracheal intubation or with difficulty responding to simple commands.

The evaluation was semi-structured and performed through an evaluation form developed according to the manual and protocols established by the Ministry of Health for the care of hypertension and diabetes mellitus [32].

### 2.6 Bias

All research participants presented a medical diagnosis of type 2 DM (DM-II) confirmed by blood glucose measurement and laboratory tests in order to avoid the emergence of possible sources of bias during data collection.

The selection bias was alleviated with the recruitment of all participants who were hospitalized for clinical follow-up in the emergency, recovery room and vascular ward sectors. All of these patients were followed by the nursing staff who informed the study researcher (FLMTA) of all new patients to be evaluated.

Deaths from DM are a source of prevalence bias, but they represented only 5% (275/5,284) of hospitalizations during the study period.

### 2.7 Quantitative variables

The outcome variable is ordinal qualitative. The 17 independent or explanatory variables are mostly nominal or ordinal qualitative variables (88%; 15 out of 17). The only two quantitative variables are: (1) age and (2) number of years after DM diagnosis. The age variable was categorized into two categories with mean age as the cut-off point (67 years). The number of years after DM diagnosis is a variable with much missing data (= 57 individuals who had no prior knowledge), and therefore was not considered in the statistical analyzes.

### 2.8 Statistical analyzes

Two-by-two (2 × 2) contingency tables were applied for exploratory analysis and selection of independent variables for the regression model considering a significance level of 20%. Robust variance Poisson regression models and step forward variable selection method were applied at a significance level of 5% for the 95% confidence interval prevalence ratios.

Three binary outcome variables were constructed: (1) outcome 1 – severe vs. outcome 0 – less severe; (2) outcome 2 – very severe vs. outcome 0; and (3) outcome 3 – extremely severe vs. outcome 0. Therefore, three multiple regression models were constructed, one for each binary outcome variable.

Programming codes were developed in the R version 3.5.1 program (*The R Foundation for Statistical Computing*; https://www.r-project.org/).

## 3 Results

### 3.1 Participants

Eligible patients with diabetes mellitus who underwent lower limb amputation were serially listed during the cumulative sampling until the pre-determined minimum sample size was reached. The serial list was cut off at the 385th patient. In other words, we evaluated 385 individuals with DM-II with LLA as the study population. In addition, we identified a total of 5,284 individuals hospitalized with DM in the *HGF* during the study period (Table 1), according to *DATASUS* estimates [33] in the Unified Health System (*SUS*).

**Table 1:** Hospital admissions and deaths from DM in the Fortaleza General Hospital (*HGF*) of the Unified Health System (*SUS*) from Feb. 2017 to Apr. 2018.

*SUS* Hospital Morbidity by DM – *Hospital Geral de Fortaleza*		
Month/Year	Number of hospitalizations	Deaths
April/2018	205	9
March/2018	362	10
February/2018	359	16
January/2018	392	18
December/2017	368	16
November/2017	354	19
October/2017	414	20
September/2017	359	12
August/2017	402	23
July/2017	332	27
June/2017	338	16
May/2017	377	25
April/2017	390	26
March/2017	356	31
February/2017	276	7
**Total**	**5.284**	**275**

Legend: *SUS*, Unified Health System. Source: *Datasus* [33].

Out of the 385 cases, we observed 10 deaths occurring from causes related to the macrovascular complications and multiple organ failure.

### 3.2 Descriptive data

The sociodemographic, clinical and epidemiological information of the sample population (n = 385) were described in Table 2.

**Table 2:** Demographic, clinical and epidemiological characterization of the sample, Fortaleza, Ceará, Brazil, 2017–2018.

Variables 1	Categories	N	%
LLA severity (outcome)	Disarticulation and bilateral (Outcome 3)	80	20.8
	Suprapatellar (Outcome 2)	107	27.8
	Transmetatarsal and infrapatellar (Outcome 1)	97	25.2
	Toe (Outcome 0)	101	26.2
Age (Years)	≥67	206	53.5
	<67 (*reference*)	179	46.5
Gender	Male	226	58.7
	Female (*reference*)	159	41.3
Education level	Incomplete	139	36.1
	Elementary	186	49.3
	Illiterate (*reference*)	60	15.6
City	Rural	172	44.7
	Fortaleza (*reference*)	213	55.3
PKDM	No	56	14.6
	Yes (*reference*)	329	85.4
Smoking	Yes	206	53.5
	No (*reference*)	179	46.5
Alcoholism	Yes	155	40.3
	No (*reference*)	230	59.7
CVD	Yes	150	39
	No (*reference*)	235	61
SAH	Yes	283	73.5
	No (*reference*)	102	36.5
Glucometer	No	184	47.8
	Yes (*reference*)	201	52.2
Daily use of Gl.	No	296	76.9
	Yes (*reference*)	89	33.1
Hypoglycemic	No	118	30.7
	Yes (*reference*)	267	69.3
Insulin	No	209	54.3
	Yes (*reference*)	176	45.7
Freq.-Primary Care	Never	117	30.4
	Rarely	55	14.3
	Regularly (*reference*)	213	55.3
Primary Care	No	263	68.3
	Yes (*reference*)	122	31.7

1 Supplementary Variable Information:(1) Age, variable age recoded to greater than and less than 67 years (mean sample age);(2) PKDM, prior knowledge of diabetes mellitus;(3) CVD, cardiovascular disease;(4) SAH, systemic arterial hypertension;(5) LLA severity, severity of lower limb amputations caused by DM;(6) Daily use of Gl., Daily use of glucometer;(7) Freq.-Primary Care, frequency of attending primary care;(8) Primary Care, specific guidance on amputation in primary care DM.

**Figure 1:**
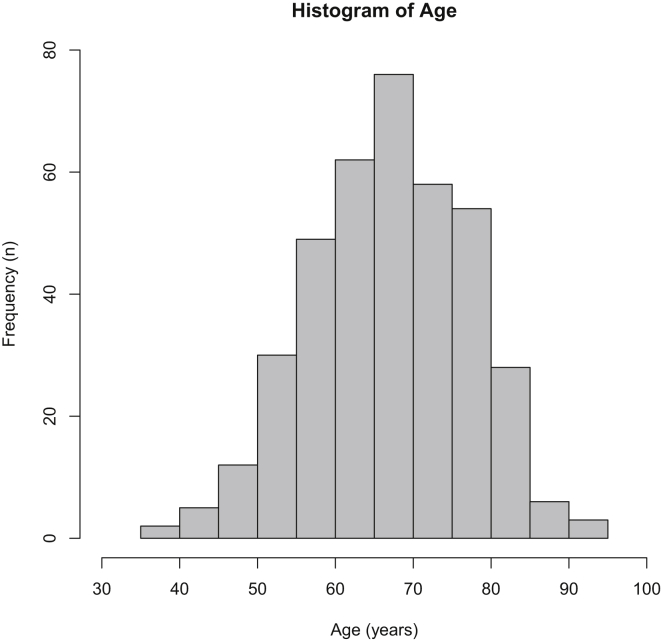
Distribution of age according to the frequency of patients in each age stratum of histogram breaks.

The prevalence of the most severe outcome (disarticulation and bilateral) in the study population was high (20.8%). The study population was characterized by predominantly male individuals with low education, residing in Fortaleza or in the interior of the state, with unhealthy habits such as smoking or alcoholism, presence of comorbidities such as arterial hypertension or cardiovascular disease, being vulnerable due to the scarce use of methods to prevent DM complications and poorly assisted in primary healthcare. In addition, the 385 cases were relatively elderly with median age = 67 and interquartile range = 60–75 years (Figure 1).

### 3.3 Exploratory data analysis

In Table 3 is shown the chi-squared (χ^2^) tests of contingency tables between: (1) clinical outcomes of amputation severity caused by DM and (2) exposure variables.

**Table 3:** Distribution of contingency tables by each exposure variable according to clinical outcome of amputation severity by diabetes mellitus, Fortaleza, Ceará, Brazil, 2017–2018.

Exposure Variables^1^	Categories	LLA Severity^2^					
		Outcome 1		Outcome 2		Outcome 3	
		1	0	2	0	3	0
Age	≥67	46	46	65	46	49	46
	<67	51	55	42	55	31	55
***χ***^**2**^		*p* = 0.902		*p* = 0.04		*p* = 0.051∗	
Gender	Male	64	49	55	49	58	49
	Female	33	52	52	42	22	52
***χ***^**2**^		*p* = 0.019		*p* = 0.781		*p* = 0.002	
Education Level	Incomplete	31	37	38	37	33	37
	Elementary	49	50	52	50	35	50
	Illiterate	17	14	17	14	12	14
***χ***^**2**^		*p* = 0.688		*p* = 0.919		*p* = 0.74	
City	Rural	40	41	52	41	39	41
	Fortaleza	57	60	55	60	41	60
***χ***^**2**^		*p* = 1		*p* = 0.307		*p* = 0.344	
PKDM	No	10	16	26	16	4	16
	Yes	87	85	81	85	76	85
***χ***^**2**^		*p* = 0.346		*p* = 0.178		*p* = 0.038	
Smoking	Yes	53	54	55	54	44	54
	No	44	47	52	47	36	47
***χ***^**2**^		*p* = 0.982		*p* = 0.25		*p* = 0.956	
Alcoholism	Yes	37	33	41	33	44	33
	No	60	68	66	68	36	68
***χ***^**2**^		*p* = 0.512		*p* = 0.481		*p* = 0.004	
CVD	Yes	30	28	58	28	34	28
	No	67	73	49	73	46	73
***χ***^**2**^		*p* = 0.735		*p* < 0.001		*p* = 0.055	
SAH	Yes	61	74	85	74	63	74
	No	36	27	22	27	17	27
***χ***^**2**^		*p* = 0.157		*p* = 0.376		*p* = 0.497	
Glucometer	No	47	52	63	52	22	52
	Yes	50	49	44	49	58	49
***χ***^**2**^		*p* = 0.776		*p* = 0.351		*p* = 0.002	
Daily use of Gl.	No	70	79	92	79	55	79
	Yes	27	22	15	22	25	22
***χ***^**2**^		*p* = 0.411		*p* = 0.2		*p* = 0.203	
Hypoglycemic	No	26	30	41	30	21	30
	Yes	71	71	66	71	59	71
***χ***^**2**^		*p* = 0.768		*p* = 0.245		*p* = 0.729	
Insulin	No	52	56	72	56	29	56
	Yes	45	45	35	45	51	45
***χ***^**2**^		*p* = 0.907		*p* = 0.107		*p* = 0.016	
Freq.-Primary Care	Never	25	25	45	25	22	25
	Rarely	13	19	13	19	10	19
	Regularly	59	57	49	57	48	57
***χ***^**2**^		*p* = 0.583		*p* = 0.026		*p* = 0.512	
Primary Care	No	60	62	86	62	55	62
	Yes	37	39	21	39	25	39
***χ***^**2**^		*p* = 1		*p* = 0.004		*p* = 0.383	

1 Supplementary information on exposure variables:(1) Age, variable age recoded to greater than and less than 67 years (mean sample age);(2) PKDM, prior knowledge of diabetes mellitus;(3) CVD, cardiovascular disease;(4) SAH, systemic arterial hypertension;(5) LLA severity, severity of lower limb amputations caused by DM;(6) Daily use of Gl., Daily use of glucometer;(7) Freq.-Primary Care, primary care attendance frequency;(8) Primary Care, specific guidance on amputation in primary care DM.

2 AMP severity, severity of amputations caused by diabetes mellitus:(1) Outcome = 0 (Toe);(2) Outcome = 1 (Transmetatarsal and Infrapatellar);(3) Outcome = 2 (Suprapatellar);(4) Outcome = 3 (Disarticulation and Bilateral).

∗ Results of chi-squared (χ^2^) tests with probability <0.2 were used as criteria for selection of exposure variables for the step of single and multiple regression models with P (outcome) ~ Poisson.

### 3.4 Main results: prevalence ratios in Poisson models

The prevalence ratios of LLA severity by DM were estimated according to the exposure variables selected in the exploratory data analysis step. Crude and adjusted estimates of prevalence ratios and their precision (i.e. 95% confidence interval) were organized by: (1) outcome = 1 (transmetatarsal and infrapatellar amputations), (2) outcome = 2 (suprapatellar amputation) and (3) outcome = 3 (disarticulated and bilateral amputations). Outcomes = 1, 2, or 3 were used as the presence of outcome, while outcome = 0 (toe amputation) was used as the absence of outcome (i.e. less severe) in all prevalence ratio estimates. Categorical and dichotomous outcome variables were use.

The main results in Table 4 were:

(1) The presence of an association between transmetatarsal and infrapatellar amputations (outcome 1) with gender (adjusted PR = 1.43, 95% CI: 1.05–1.95). Accordingly, the prevalence of outcome 1 was 43% higher in males compared to females, adjusted for arterial hypertension levels.
(2) The presence of an association between suprapatellar amputations (outcome 2) and the presence of cardiovascular disease (adjusted PR = 1.7, 95% CI: 1.32–2.19) and the absence of specific guidance on amputation in diabetes mellitus in primary care (adjusted PR = 1.52, 95% CI: 1.05–2.21). The prevalence of outcome 2 was 70% higher in individuals with cardiovascular disease and 52% higher in individuals with no primary care guidance compared with those without the disease and with primary care guidance, respectively. The estimated prevalence ratio was adjusted for age, prior knowledge of the disease and insulin use.
(3) The association between more severe amputations (outcome 3, with disarticulation and bilateral) at older age (adjusted PR = 1.55, 95% CI: 1.12–2.14), male gender (adjusted PR = 1.83, 95% CI: 1.27–2.64), alcoholism (adjusted PR = 1.70, 95% CI: 1.25–2.33), insulin use (adjusted PR = 0.68, 95% CI: 0.48–0.97). The prevalence of outcome 3 was 55% higher in longer-lived subjects, 83% higher in male subjects, 70% higher in individuals with alcoholism compared to the reference groups (assumed to be unexposed - see Table 2). The prevalence of outcome 3 was 32% lower for subjects taking insulin compared with the reference groups. Prevalence ratios were adjusted by previous knowledge of DM-II and cardiovascular disease.

**Table 4:** Poisson model results for transmetatarsal and infrapatellar amputations (outcome 1), suprapatellar amputations (outcome 2) or disarticulation and bilateral amputations (outcome 3) vs. toe (outcome 0) as a function of exposure variables.

Model	Variables	PR (95%CI)	P (*z*)
**Outcome 1 vs. 0**			
Univariate	Gender	1.46 (1.07–1.99)	0.018∗
Univariate	SAH	0.79 (0.6–1.05)	0.104
Bivariate	Gender	1.43 (1.05–1.95)	0.025∗
	SAH	0.82 (0.62–1.09)	0.178

**Outcome 2 vs. 0**			
Univariate	Age	1.35 (1.03–1.78)	0.032∗
Univariate	PKDM	1.27 (0.96–1.69)	0.1
Univariate	CVD	1.68 (1.29–2.18)	<0.001∗
Univariate	Insulin	1.29 (0.96–1.72)	0.09
Univariate	Freq.-Primary Care	1.18 (1.02–1.36)	0.02∗
Univariate	Primary Care	1.66 (1.15–2.41)	0.007∗
Multiple1: All Exposure Variables	Age	1.26 (0.97–1.63)	0.08
	PKDM	0.95 (0.68–1.32)	0.76
	CVD	1.76 (1.37–2.27)	<0.001∗
	Insulin	1.06 (0.77–1.45)	0.725
	Freq.-Primary Care	1.15 (0.96–1.37)	0.13
	Primary Care	1.41 (0.96–2.09)	0.082
Multiple 2: No FreqPC (collinear with PC)	Age	1.26 (0.97–1.64)	0.08
	PKDM	1.1 (0.83–1.46)	0.51
	CVD	1.7 (1.32–2.19)	<0.001∗
	Insulin	1.1 (0.81–1.50)	0.55
	Primary Care	1.52 (1.05–2.21)	0.028∗

**Outcome 3 vs. 0**			
Univariate	Age	1.43 (1.02–2.02)	0.04∗
Univariate	Gender	1.82 (1.23–2.7)	0.001∗
Univariate	PKDM	0.42 (0.17–1.03)	0.06
Univariate	Alcoholism	1.65 (1.19–2.29)	0.003∗
Univariate	CVD	1.42 (1.03–1.95)	0.032∗
Univariate	Insulin	0.64 (0.45–0.91)	0.013∗
Multiple1: All Exposure Variables	Age	1.58 (1.14–2.19)	0.006∗
	Gender	1.51 (0.98–2.4)	0.064
	PKDM	0.51 (0.22–1.19)	0.112
	Alcoholism	1.34 (0.98–1.95)	0.118
	CVD	1.17 (0.85–1.60)	0.332
	Insulin	0.67 (0.47–0.95)	0.023∗
Multiple 2: No Alcoholism (collinear with Gender)	Age	1.55 (1.12–2.14)	0.008∗
	Gender	1.83 (1.27–2.64)	0.001∗
	PKDM	0.49 (0.21–1.15)	0.1
	CVD	1.15 (0.84–1.59)	0.381
	Insulin	0.68 (0.48–0.97)	0.031∗

Legend: PR, prevalence ratio; CI, confidence interval; P, p-value.

Age, variable age recoded greater than and less than 67 years old; PKDM, previous knowledge about diabetes mellitus; CVD, cardiovascular disease; Freq.-Primary Care, frequency of primary care assistance; Primary Care, specific guidance on amputation in diabetes mellitus in primary care.

∗ Results of z-statistic tests with probability <0.05 confirm the statistical and significant association between exposure variable and outcome = 1, 2 or 3.

## 4 Discussion

### 4.1 Main results

In the present study, a prevalence of 7% (study sample = 385/accessible population = 5,284) of lower limb amputations was identified in patients with DM in the *HGF*. Additionally, the prevalence of LLA severity degrees in DM-II varied as follows: (1) less severe outcome (toe), prevalence = 1.5% (80/5,284); (2) severe outcome (transmetatarsal or infrapatellar), prevalence = 2% (107/5,284); (3) very severe (suprapatellar) outcome, prevalence = 1.8% (97/5,284); and (4) extremely severe outcome (disarticulation or bilateral), prevalence = 1.9% (101/5,284). The prevalence ratio analysis of the degrees of LLA severity showed the following associated factors: (1) demographic (male gender and longevity), (2) clinical (presence of cardiovascular disease and insulin use), (3) epidemiological (alcoholism), and (4) access to specific guidance in primary healthcare.

### 4.2 Interpretations

DM is a serious disease, which is frequent in the adult population, representing almost 5% of the disease burden in Brazil [34]. In the present study, it was observed that 58% of the individuals hospitalized with DM who suffered LLA in the hospital environment were male. About 53% of the sample was 67 years of age or older and 65% had low educational level. In a prevalence study conducted in a public hospital in Recife with 107 individuals with diabetic foot who suffered amputations, the identified sociodemographic characteristics were: (1) average age of 65 years, (2) higher involvement in males and (3) low education level [35]. The Fremantle cohort study conducted in Australia with individuals with DM-II who underwent LLA showed a mean age of 64–65 years and greater involvement for males [36]. The Brazupa study found a higher proportion of amputations in older male patients with a longer DM diagnosis [25]. The literature also shows an increased incidence of amputations in individuals with DM from 60 years of age [37] and population aging directly implies in this incidence, especially in males [38, 39, 40]. In hospital-based studies, the 6^th^ and 7^th^ decades of life are associated with a higher occurrence of diabetic foot and amputations [34, 40]. All these studies corroborate the results found in our research.

*HGF* is a reference unit in the state of Ceará and offers highly complex clinical care for patients with DM. It was observed that 44.7% of the sample in the present study were from the city of Fortaleza, while 55.3% of the individuals evaluated were from the interior of the state. Thus, the *HGF* provided clinical support to 65 municipalities; therefore reinforcing the role of this hospital as a reference center for DM care in the metropolitan region of Fortaleza and in cities in the interior of the state.

Approximately 15% of the sample had no previous knowledge about DM and these patients were admitted to the hospital without knowing the clinical diagnosis of the disease, which directly affected the LLA. As DM is a silent condition, the absence or delay in the initiation of adequate treatment can lead to its progression and severe outcomes as a consequence [41]. In the present study, it was observed that 77% of the sample did not perform daily blood glucose control and 48% did not have a personal monitoring glucometer. The regular control of blood glucose levels is a major challenge for most diabetic individuals due to the difficulty in measuring, lack of resources to acquire equipment and difficulties in handling the equipment. Additionally, the high cost of purchasing disposable biosensor tape restricts its wide use. Glycemic control significantly reduces the risk of macro and microvascular complications of DM-II [42]. Hyperglycemia induces intense pathological processes, dysfunctions and insufficiency in various organs and tissues in the long term, compromising the autonomy and quality of life of individuals with diabetes [42].

LLA is a last resort intervention for the consequences of DM [25]. It was observed that the severity of suprapatellar amputation (27.8%) was the most commonly performed in the hospital environment in the present study. Chan et al. (2009) [37] investigated 140 individuals amputated by DM, noting that 63.3% of these procedures were performed on the toes (phalanges, toe bones, tarsus, metatarsus or foot). In a 3-year retrospective study, Toursarkissian et al. (2002) [43] identified 56% of primary LLAs in 99 men with a 3:2 ratio for above/below knee amputation level. Spichleret al. (2004) [38] found 28% of the sample (1,390 individuals with DM) who suffered thigh-level amputation.

Suprapatellar amputation correlated with the presence of cardiovascular disease, insulin use, and low primary healthcare attendance with specific guidance. Disarticulation and bilateral amputations were associated with age ≥67 years, male gender, alcoholism, and insulin use adjusted by previous knowledge of DM, cardiovascular disease, and specific primary healthcare guidelines.

These results directly reflect the relationship between DM and risk factors and cardiovascular changes. The use of insulin affected the occurrence of the most severe, suprapatellar, disarticulation and bilateral amputations. This may be directly related to poor glycemic control of the disease, poor adjustments in administering adequate insulin dosage for each patient, inadequate food management and lack of physical activity. Associated with these possible factors, low self-monitoring with glucometer use observed in this study may have enhanced these results, indicating that most of the evaluated individuals did not perform precise monitoring of glycemic level, increasing the risk of complications. Approximately 74% of the study sample had hypertension. This change occasionally contributes to the development and progression of chronic complications of DM [44], and is part of a syndrome which includes glucose intolerance, insulin resistance, obesity, dyslipidemia, and coronary artery disease. These are intervening factors for the formation of neuroischemic ulcers and amputations [45].

The low frequency and assistance in primary healthcare imply a scarce approach to pathology and health education strategies to guide the population. The low educational level of individuals with DM associated with the low support of health education by primary healthcare observed in this study directly reflects the incidence of these amputations. The limited amount of information on healthcare, prevention measures, habits and risk behaviors may make this population segment more likely to develop such complications.

Economic, educational and social differences play an important role together with disparities in access to health, the occurrence and severity of amputations [25]. Santos et al. (2013) [35] analyzed the prevalence of diabetic foot amputations in Pernambuco and found that low education, two or more people living in the household, income below one minimum monthly salary, lack of foot evaluation, did not receive guidance on care for feet in consultations in the past year, not using medication to control DM and inadequate control of blood glucose were all factors associated with the occurrence of amputations. Increased awareness among health professionals and faster access to specialist centers may result in increased diagnosis, leading to improved prognosis and treatment [25].

The lack of support to health services due to the ineffectiveness of health policies, which do not guarantee equity in DM treatment and health education, are risk factors for worsening of the pathology [44]. The high incidence of amputations due to diabetes complications reflects the high prevalence of the disease, limited resources in care and late treatment [46]. The inefficiency of early diagnosis, especially in the primary care network, leads to underreporting of intermittent claudication, increasing the risk of ischemic events, gangrene, amputation and death [38, 39]. In addition, the diagnosis is ineffective because approximately half of individuals with DM do not know its condition, and 20% of those who know it do not undergo any treatment [37]. In these individuals, ulcers and tissue lesions are very likely to become infected due to lack of self-care and guidance. Individuals with these complications are consequently often seen in the emergency department and undergo primary amputations as the only possible treatment [37].

Early preventive measures have been reported by several studies as fundamental for amputation prevention [34, 35], and most of the risk factors for amputations are amenable to primary prevention with the support of adequate health care [44]. Despite advances in primary healthcare over the years, amputations in individuals with DM are still a very common reality in Brazil and the state of Ceará. Thus, it is necessary to modify, expand and intensify public healthcare policies for patients with DM, implement educational strategies and reorganize surveillance services and comprehensive healthcare at all levels of complexity to promote care. clinical and educational treatment for diabetic patients.

### 4.3 Limitations

This research has some limitations, namely: (1) no evaluation of clinical conditions prior to amputations was performed; (2) there is no information on the precise dosage of insulin use. This is because inadequate dosing (i.e. hypodose) could have contributed to hyperglycemia peaks; (3) there was no clinical follow-up through reevaluation during the outpatient care segment of amputated patients; and (4) the possibility of reverse causality which may occur through cross-sectional studies.

## Declarations

### Author contribution statement

F. Alves: Conceived and designed the experiments; Performed the experiments; Analyzed and interpreted the data; Contributed reagents, materials, analysis tools or data; Wrote the paper.

G. Laporta: Conceived and designed the experiments; Analyzed and interpreted the data; Contributed reagents, materials, analysis tools or data; Wrote the paper.

### Funding statement

G. Laporta was supported by 10.13039/501100003593Conselho Nacional de Desenvolvimento Científico e Tecnológico (307432/2019-0).

### Competing interest statement

The authors declare no conflict of interest.

### Additional information

No additional information is available for this paper.
